# A rare case of retroperitoneal teratoma with evidence of papillary thyroid carcinoma: a case report

**DOI:** 10.1186/s12902-024-01606-4

**Published:** 2024-06-10

**Authors:** Adisa Poljo, Jennifer M. Klasen, Marco von Strauss und Torney, Alberto Posabella, Stephanie Taha-Mehlitz, Barbara Hummer, Beatrice Kern

**Affiliations:** 1 University Digestive Health Care Center Basel - Clarunis, Department of Visceral Surgery, St. Claraspital and University Hospital Basel, Basel, 4002 Switzerland; 2 St. Clara Research Ltd, Basel, 4058 Switzerland; 3 Department of Clinical Pathology and Cytopathology, Viollier AG, Allschwill, 4123 Switzerland

**Keywords:** Case report, Retroperitoneal teratoma, Papillary thyroid carcinoma, Malignant struma ovarii

## Abstract

**Background:**

Teratomas are germ cell tumors composed of somatic tissues from up to three germ layers. Primary retroperitoneal teratomas usually develop during childhood and are uncommon in adults and in the retroperitoneal space. While there are only a few cases of retroperitoneal thyroid tissue, we report a unique case of a retroperitoneal papillary thyroid carcinoma.

**Case presentation:**

A 41-year-old woman presented in our institution due to intermitted unspecific abdominal pain. Magnetic resonance imaging detected a multi-cystic solid retroperitoneal mass ventral to the psoas muscle and the left iliac artery. After surgical removal of the retroperitoneal mass, histology sections of the specimen indicated evidence of papillary thyroid carcinoma cells. A staging computed tomography scan of the body showed no further manifestations. To reduce the risk of recurrence, total thyroidectomy was performed followed by radioiodine therapy with lifelong hormone substitution.

**Conclusions:**

Primary retroperitoneal teratoma with evidence of papillary thyroid carcinoma is a rare condition. Preoperative diagnosis is difficult due to its non-specific clinical manifestation and lack of specific radiologic findings. Histopathology analysis is necessary for diagnosis. Although surgery is considered the first line treatment, there is still discussion about the extent of resection and the need for total thyroidectomy with adjuvant radioiodine therapy.

## Background

A teratoma is a germ cell neoplasm mainly composed of different tissues, which do not correspond to the tissue in which it occurs. It can be benign or malignant and is classified as mono-, bi-, or tri-dermal (ectoderm, mesoderm, or endoderm), depending on the number of germ layers present. Teratomas occur more commonly in children but can also be found in adults in different locations [1]. Although primary teratomas in adults are rare, accounting for only 4% of all primary teratomas [2], the retroperitoneal space stands as the fourth most common site of teratoma origin, following the ovaries, testis, and anterior mediastinum [3].

The clinical manifestations of this disease are non-specific. Case reports make up the majority of the literature [1, 4]. Given the absence of specific imaging signs, achieving a preoperative diagnosis is notably challenging, necessitating pathological examination for confirmation. Considering additional imaging features, including the detection of fat tissue in computed tomography (CT) or magnetic resonance imaging (MRI) and the identification of gross calcifications, proves instrumental in refining the list of potential differential diagnoses. Moreover, other notable findings, such as complex cystic structures, heterogeneous tissue composition, or the presence of distinct tissue types, can further enhance diagnostic specificity in the case of teratomas [5].

Herein, we report a case of a retroperitoneal teratoma with evidence of papillary thyroid carcinoma. To our knowledge, this is the first case report presenting primary retroperitoneal papillary thyroid cancer.

## Case presentation

A 41-year-old woman was admitted to the hospital with a clinical history of recurrent lower abdominal pain.

She had a past medical history of epilepsy since the age of 17, currently under treatment, obesity WHO grade I and arterial hypertension. Ten years ago, she underwent a removal of an ovarian cyst, whereby the side remained unclear. Her sister was suffering from systemic sclerosis and her uncle was diagnosed with lung cancer. In a previous gynecological sonography report a cystic lesion measuring 44 × 27 × 30 mm was described which could be differentiated from the adnexal ligament and was located cranially to the uterus.

At the time of admission, the patient was asymptomatic, and both her physical examination and vital signs were unremarkable. The abdomen presented soft, and not distended with normoactive bowel sounds. There were no signs of hepatosplenomegaly or any masses.

**Fig. 1:**
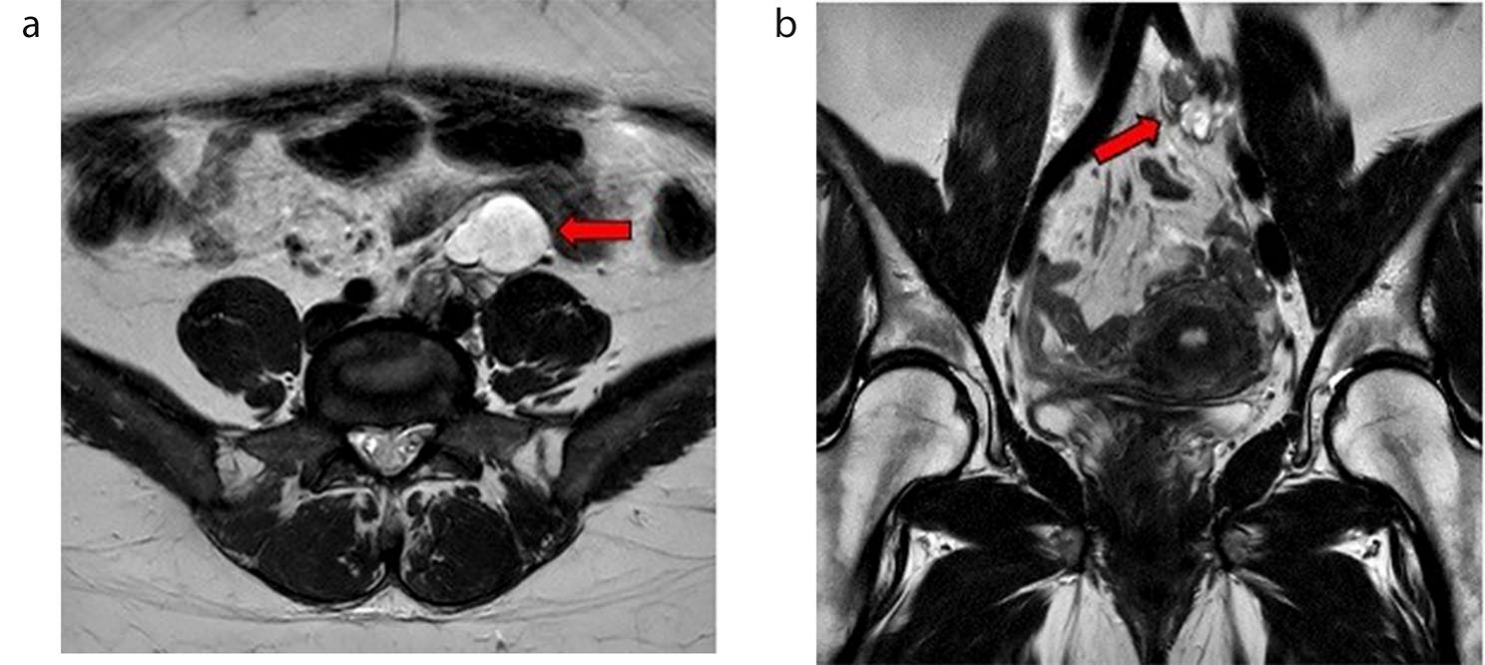
Abdominal MRI images in T2W/TSE-tra (**a**) and cor (**b**) showing **a** multicystic solid retroperitoneal mass ventral to the psoas muscle and the left iliac artery (red arrow) **b** lobulated, partly cystic and partly solid portion with vascular contact (red arrow)

Because of the patient’s young age MRI was performed and showed a multicystic solid retroperitoneal mass ventral to the psoas muscle and the left iliac artery with a polycystic portion of 37 × 26 × 45 mm ventrally (Fig. 1a), and a lobulated, partly cystic and partly solid portion of 36 × 15 × 33 mm dorsally (Fig. 1b) with vascular contact, ultimately of unclear etiology. Due to the solid part of the tumor, a malignant aspect was considered with the fatty part being indicative of a retroperitoneal teratoma. Differentially, cystic mesothelioma, mucinous cystadenoma, and lymphatic malformation were also considered but deemed unlikely due to the solid parts of the mass. No connection to the ovary or parenchymal bridge of the presumed horseshoe kidney was detected.

After an interdisciplinary discussion of the radiological findings, surgical removal of the retroperitoneal mass was recommended.

A diagnostic laparoscopy was performed. After locating and sparing the iliac vessels and the left ureter, mobilization of the sigmoid colon and left flexure was performed. Due to adhesions between the tumor mass and the iliac vessels, it was necessary to convert to a laparotomy. The tumor mass was then completely removed. The patient’s postoperative course was uneventful and she was discharged within three days.

**Fig. 2:**
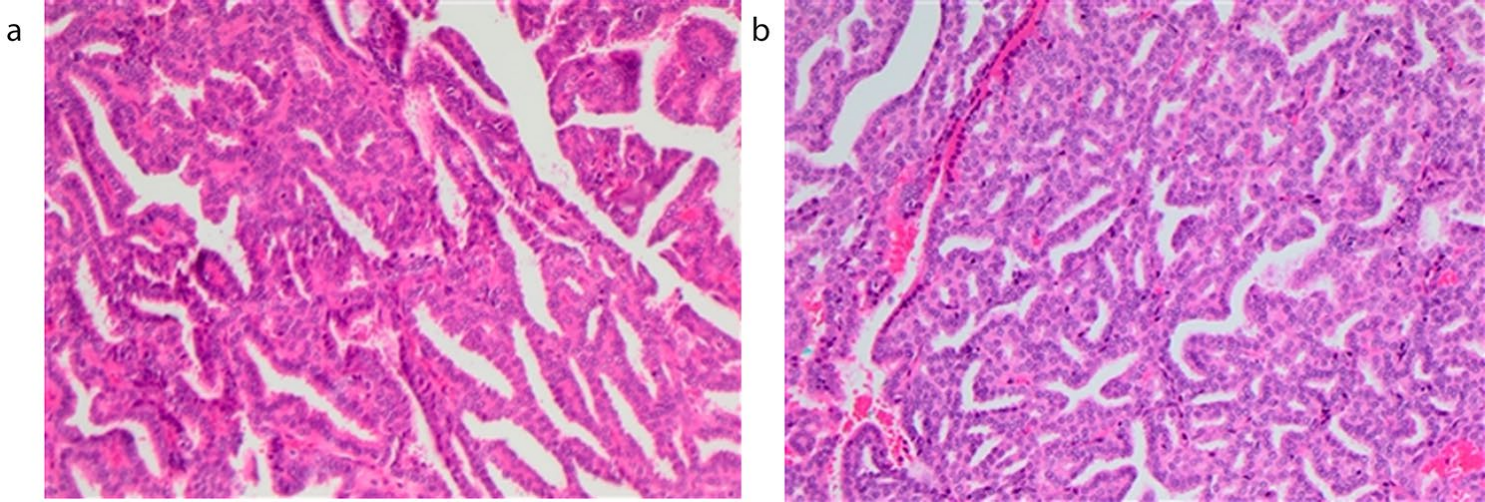
**a**, **b** Surgically resected specimen of case. Hematoxylin and Eosin (H&E) staining; Papillary carcinoma in a retroperitoneal teratoma

Histological workup revealed fragmentary retroperitoneal teratoma with evidence of a papillary thyroid carcinoma of 1.4 cm in one of the fragments (Fig. 2a and b) and consistent nuclear expressions of PAX8 and TTF-1. There was no evidence of lymphangiosis or hemangiosis carcinomatosa and no perineural sheath infiltrations. Resection in toto (minimal distance to the surface of the affected fragment 0.01 cm) was confirmed.

Considering the histological results, further staging was completed. A thyroid sonography showed neither a pathological finding in the gland nor cervical lymphadenopathy. An enhanced chest CT encompassing the neck, thorax, and abdomen was conducted, indicating no evidence of tumor manifestations in the cervicothoracic or abdominal regions.

Tumor markers for a malignant germ cell tumor and thyroid function parameters were all within the normal range.

As this retroperitoneal teratoma behaved like a malignant struma ovarii (MSO), it was re-discussed in both, the endocrinological and gynecological department whether an adnexectomy should be recommended on the left side. Gynecological examination and vaginal sonography revealed no suspicious finding, while both ovaries presented unremarkable with normal follicular structure in the ultrasound. There was no evidence of other space-occupying lesions in the lesser pelvis and no free fluid in the Douglas space.

From a gynecological point of view, there was no indication of unilateral adnexectomy because the retroperitoneal teratoma was removed from a different compartment and distant from the inconspicuous left ovary.

Since no evidence-based opinion about the benefit of such an operation exists yet, it was intensively discussed with the patient and agreed to refrain from surgical removal of the adnexa and to continue with regular monitoring of the ovaries. Regarding the thyroid carcinoma in the teratoma with increased risk due to the size, total thyroidectomy and subsequently a radioiodine therapy were recommended in analogy to the same carcinoma of the thyroid gland, whereby a lifelong hormone substitution would be necessary afterwards.

Therefore, a complete thyroid gland removal was discussed and scheduled upon consent.

The neck exploration intraoperatively showed a macroscopically inconspicuous thyroid gland on both sides. Both thyroid lobes were removed in toto. The patient tolerated the procedure well and was able to leave the hospital after a hospitalization of two days.

Histology revealed diffuse normo-follicular colloid struma without evidence of dysplastic or malignant changes. Postoperatively, sonographic and scintigraphic examinations revealed only minimal residual tissue in the right thyroid lobe. In the radioiodine test, the maximum retention value was approximately 1.18% of the administered activity. With only minimal residual tissue, radioiodine elimination with 131-Iodine was possible and was scheduled five weeks after thyroidectomy. Three months after ablative therapy, both the clinical examination and laboratory results showed no indications of tumor recurrence or metastasis, with undetectable levels of the tumor marker thyroglobulin (Tg < 0.1 µg/L (reference values < 0.1), Tg-antibodies 15 IU/l (reference values < 115). Nine months after thyroidectomy, an extended tumor follow-up examination was conducted, including ablation control through 123-Iodine whole-body scintigraphy and SPECT/CT after recombinant human thyrotropin (rhTSH) stimulation. Fortunately, once again, no tumor recurrence was observed.

## Discussion

Primary retroperitoneal teratomas in adults are uncommon tumors that typically appear in children. In 1937 the first described case of retroperitoneal teratoma was determined radiologically with urography [6]. There are two forms of teratomas on a macroscopic level: solid teratomas, which are typically malignant and made of fibrous tissue, fat, cartilage, bone, and undeveloped embryonic tissue, and cystic teratomas, which are typically benign and made of completely developed elements [4]. Additionally, teratomas are divided into two histologic types: mature and immature. Mature teratomas contain elements that have undergone complete somatic differentiation, whereas immature teratomas contain elements with only partial somatic differentiation similar to embryonic or fetal tissue [1].

Diagnosing a retroperitoneal teratoma remains challenging, as there are no specific radiological criteria. Retroperitoneal teratomas can be predominantly cystic or entirely solid in form. In more than half of patients, plain abdomen films show a tissue mass with calcifications which can appear irregularly shaped within the neoplasm or arciform encircling all or part of the surface. Adipose tissue, sebaceous and serous-type fluid, as well as soft tissue density structures, can be detected using CT to diagnose these neoplasms [1]. Although CT-guided biopsy was not part of the evaluation, biopsy may be useful in the diagnosis of such cases. However, it may not sample all areas, thus missing immature or malignant tissues that may be present [7, 8]. With the aid of cross-sectional imaging studies, the relationship of the tumor mass to adjacent tissue and organ structures can be depicted more precisely. MRI allows better visualization of the different components of the teratoma as well as possible invasion of blood vessels and nerves. Sarcomas, lymphomas and tumors originating from adjacent organs (e.g. pancreas, kidney, adrenal gland etc.) should also be considered as differential diagnosis [1].

The occurrence of malignant thyroid tissue is known in the teratoma of the ovary as so-called malignant struma ovarii (MSO). Struma ovarii makes up to 3% of all ovarian teratomas and contains thyroid tissue in more than 50% [9, 10]. It was first described by Von Kalden in 1895 and Gottschalk in 1899 [11] and can occur in women of any age but with a peak incidence in the fifth decade of life [12] and is less common in postmenopausal women [13]. Usually, this tumor is benign with a malignant transformation rate of only 5%. Clinical symptoms are not specific and include abdominal symptoms like stomach pain and irregular vaginal bleeding [14].

Due to the rarity of MSO, there is still very few data about this condition, with the vast majority coming from case reports. Therefore, no appropriate treatment concept is available yet. Most experts recommend abdominal hysterectomy, bilateral salpingo-oophorectomy with omentectomy, and unilateral adnexal resection for young patients without metastases in order to maintain fertility as much as possible. Comparable to our case, extra ovarian tumor extension is seen as a reason for total thyroidectomy and adjuvant treatment like radioactive iodine I-131 ablation in postoperative patients. Before initiating radioiodine therapy, it is crucial to ensure the complete removal of intact thyroid tissue. Failure to do so may result in the carcinoma tissue storing radioiodine to a lesser extent. Consequently, there would be insufficient accumulation in the residual tumor tissue, impeding the effectiveness in preventing recurrences or metastases [15]. The most extensive debate has been about whether or not patients without signs of metastases should regularly receive adjuvant therapy. While some studies have suggested no relevant differences in the recurrence rate and prognosis of patients despite the use of adjuvant therapy after surgery, others report lower recurrence rates in patients who received routine adjuvant therapy than in patients who underwent only surgery [16–19]. When retroperitoneal teratomas contain malignant tissue, particularly germ cell carcinomas, adjuvant chemotherapy and radiotherapy are required. Malignant teratomas have a 61% overall response rate being very resistant to radiation and chemotherapy. To find early recurrence, continuous post-operative follow-up is recommended [20, 21].

Although the clinical course is long and strenuous, most patients show a good prognosis and achieve a long survival duration after treatment, even in the presence of metastases. An exception is the histological subtype of atypical papillary thyroid carcinoma, which is an independent predictor of poor prognosis and treatment failure [22].

The unique finding of the described case was the missing involvement of the ovary. Therefore, it cannot be defined as MSO and represents exceptional evidence of papillary thyroid carcinoma located in the retroperitoneal space.

Because of the great uncertainty that still prevails in the treatment of retroperitoneal teratomas, more evidence is required to make specific treatment recommendations and to assess the quality of life and morbidity associated with adjuvant therapies.

## Conclusion

In summary, papillary carcinoma arising from a primary retroperitoneal teratoma stays infrequent. As the clinical manifestations are non-specific, the preoperative diagnoses remain challenging. Histopathology is inevitable to verify the diagnosis. The available evidence is limited to case reports and series and therefore subject to selection bias. Treatment decisions therefore need to be made and balanced for individual patients taking into account their age, life expectancy and willingness to take the risks of invasive treatments against the risks of an uncertain but possibly progressive disease.

## Data Availability

All the data generated and/or analyzed during this study are included in this published article.

## Abbreviations

*AFP*: *alpha-fetoprotein*
*CT*: *computed tomography*
*fT3*: *free triiodothyronine*
*fT4*: *free thyroxine*
*hCG*: *human chorionic gonadotropin*
*H&E*: *hematoxylin and eosin*
*PAX8*: *paired-box gene 8*
*LDH*: *lactate dehydrogenase*
*MRI*: *magnetic resonance imaging*
*MSO*: *malignant struma ovarii*
*Tg*: *thyreoglobulin*
*TSH*: *thyroid stimulating hormone*
*TTF-1*: *thyroid transcription factor 1*
